# Emerging Role of Exosomes in Retinal Diseases

**DOI:** 10.3389/fcell.2021.643680

**Published:** 2021-04-01

**Authors:** Zhengyu Zhang, Aime Mugisha, Silvia Fransisca, Qinghuai Liu, Ping Xie, Zizhong Hu

**Affiliations:** Department of Ophthalmology, The First Affiliated Hospital of Nanjing Medical University, Nanjing, China

**Keywords:** exosome, extracellular vesicles, retina, stem cell, miRNAs

## Abstract

Retinal diseases, the leading causes of vison loss and blindness, are associated with complicated pathogeneses such as angiogenesis, inflammation, immune regulation, fibrous proliferation, and neurodegeneration. The retina is a complex tissue, where the various resident cell types communicate between themselves and with cells from the blood and immune systems. Exosomes, which are bilayer membrane vesicles with diameters of 30–150 nm, carry a variety of proteins, lipids, and nucleic acids, and participate in cell-to-cell communication. Recently, the roles of exosomes in pathophysiological process and their therapeutic potential have been emerging. Here, we critically review the roles of exosomes as possible intracellular mediators and discuss the possibility of using exosomes as therapeutic agents in retinal diseases.

## Introduction

**FIGURE 1:**
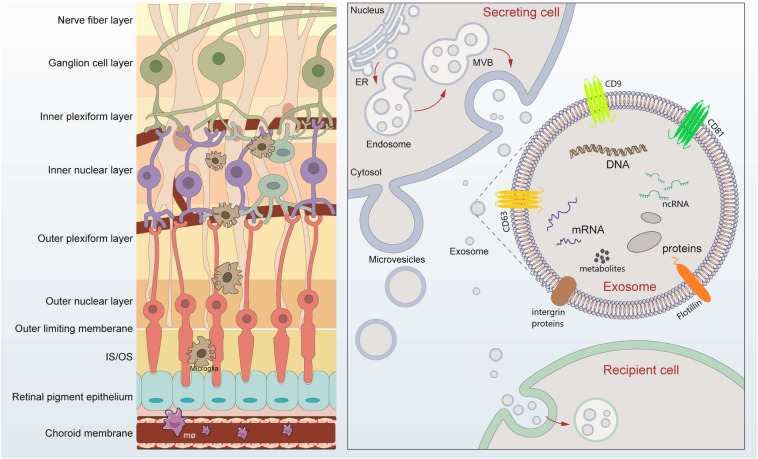
Retinal layers and schematic signaling transferred by exosomes. Retina can be roughly divided into nine layers, which tightly contact lay by lay. Exosomes are released after fusion of multivesicular bodies (MVBs) with the plasma membrane. The lipid bilayer of exosomes contains specific lipids such as cholesterol and phosphatidylserine, which protect the contents from degeneration. The cargos loaded by exosomes includes microRNAs, long-non-coding RNAs, DNA, message RNA, lipids, metabolites, and proteins, mediating possible exchange among different retinal cells. Exosomes of different cellular origin share certain common protein components such as CD9, CD81, and CD63.

Retina, is essential for performing the first stages of image processing. Multiple retinal diseases, such as age-related macular degeneration (AMD), diabetic retinopathy (DR), retinal detachment (RD), and glaucomatous neuropathy, involve angiogenic, neurodegenerative, inflammatory, and fibrotic changes which disrupt the normally transparent retina and cause irreversible vision loss (Flaxman et al., 2017). As part of central nervous system, the retina is composed of neurons, glias, endothelial cells, pericytes, immune cells, and extracellular matrix components (Figure 1). However, questions remain unveiled concerning which changes occur in these individual cells and how they interplay with each other in diseased retinal microenvironment. Deeper understanding of the cell-to-cell communication and molecular changes in retinal diseases is improvement for future preventative and therapeutic strategies.

Extracellular vesicles (EVs) are a general term for secretory vesicles, which can be classified as exosomes, microvesicles, or apoptotic bodies according to the size, surface proteins, and internal cargos. The exosome, usually ranging from 40 to 160 nm in diameter, originates from the inward budding of the plasma membrane (Figure 1) and plays an important role in intercellular communication *via* cargo molecules. Biogenetically, the extracellular components (such as RNAs, proteins, lipids, metabolites, small molecules) and the cell surface proteins sprout inward through the invagination of the plasma membrane, forming the early-sorting endosomes (ESEs). ESEs then gradually matured into late-sorting endosomes (LSEs), and transformed to multivesicular bodies (MVBs) through the inward invagination of the endosomal limiting membrane (Alenquer and Amorim, 2015; Gurunathan et al., 2019). If not fusion with lysosomes that degrade their cargo, the MVBs will be transported to the plasma membrane and released into extracellular environment as exosomes. The lipid bilayer of exosomes contains specific lipids such as cholesterol and phosphatidylserine, which protect the contents from degeneration (Figure 1; Alenquer and Amorim, 2015; Gurunathan et al., 2019). During the past decade, exosomes have attracted increasing attentions and emerged as a promising field of biomedical research for a range of diseases. First, exosomes have been suggested as biomarkers for diagnosing diseases (Howard et al., 2020; Xiao et al., 2020). Second, cargos within the exosomes participate in cell-to-cell communication through receptor-ligand interactions with target cells (Becker et al., 2016; van Niel et al., 2018). Third, exosomes, in the form of drug delivery vehicles, have the great potential to develop as therapeutic tools for a variety of currently incurable diseases (Xu R. et al., 2018; Liu and Su, 2019).

The exosomes, shed by various ocular cell type, can exist extensively in ocular fluid such as tears, aqueous humor, vitreous humor, and blood. Although the roles of various cell-derived exosomes have been increasingly investigated in diverse ophthalmic diseases, knowledge regarding the function of exosomes in retinal diseases remains limited. Here, we performed an extensive literature review to present the latest information regarding the separation strategies of exosomes, cargos loaded in exosomes, and more importantly, the role of exosomes in retinal diseases and their potential applications as therapeutic tools.

## Cargos Loaded in Exosomes

### Mechanism of Cargos Loaded Into Exosomes

Most of the reports concerning cargos loaded in exosomes have focused on the non-coding RNAs, including miRNA, lncRNA, and circRNA. To investigate the components in exosomes, the first step is to efficiently separate exosomes from biological fluids. Table 1 shows the details of the mostly used strategies at present.

**TABLE 1:** Strategies for exosome separation.

**Isolation strategy**	**Principle**	**Advantages**	**Disadvantages**
Differential ultracentrifugation	Particles with different density, size and mass show different deposition rates under centrifugal force.	• Suitable for mass preparation • Low cost	• Centrifuge equipment needed • Time-consuming • Risk of contamination
Density-gradient separation	Exosomes further separated by density in density gradient media	• High purity	• More complex operations • Time-consuming
Ultrafiltration	Selective separation of exosomes with specific particle size by using a filter membrane with a specific molecular weight interception value	• Low cost • Fast procedure • High purity of products	• Exosome membrane blockage • Exosome deformation
Size exclusion chromatography	Particles are eluted if larger than pore size of the porous polymer	• High purity of products • Fast procedure • Suitable for blood plasma • Maintain the shape of exosome	• Low output • Limited in large samples.
Co-precipitation	The highly hydrophilic polymer interacts with the water molecules around the exosome to reduce the solubility of exosomes, resulting in precipitation	• Easy to use • Low equipment requirement • Fast procedure	• Protein pollution • Require complicated clean-up steps
Immunoaffinity capture	Based on the specific binding of exosome surface protein markers to the corresponding antibodies	• Isolation of exosomes from specific sources • High purity of products	• High-cost antibodies • Low output

In 2007, Valadi et al. (2007) discovered the existence of mRNA and miRNA in exosomes for the first time, and found that exosomes can transport mRNA and miRNA to receptor cells. Subsequently, Goldie et al. (2014), found that the proportion of miRNA isolated from the exosome was higher than that of the parent cells, suggesting that the entry of miRNA into the exosome may have a special sorting mechanism. Based on previous studies, there are four possible pathways of sorting mechanism of miRNA into exosomes as (1) the neural sphingomyelinase 2 (nSMase2)-dependent pathway (Guduric-Fuchs et al., 2012); (2) the miRNA motif and sumoylated heterogeneous nuclear ribonucleoproteins (hnRNPs)-dependent pathway (Villarroya-Beltri et al., 2013); (3) the 3′-end of the miRNA sequence-dependent pathway (Koppers-Lalic et al., 2014); and (4) the miRNA induced silencing complex (miRISC)-related pathway (Frank et al., 2010).

In the research of Qu et al. (2016), it was also found that sumoylated hnRNPA2B1 could specifically encapsulate LncARSR into exosomes. Li et al. (2015) speculated that the sorting mechanism of circRNA may be associated with the changes of related miRNA levels in production cells.

As for protein sorting, studies have shown that the endosomal sorting complex required for transport (ESCRT) family plays an important role in protein synthesis and sorting into exosomes (Juan and Fürthauer, 2018). Meanwhile, other voices also proposed the protein sorting is independent of ESCRT family (de Gassart et al., 2003; Gangalum et al., 2011). de Gassart et al. (2003) highlighted that the presence of lipid microdomains in exosomal membranes participates in exosome formation and structure. When lipid rafts is destroyed, the secretion of exosome containing protein is then inhibited (de Gassart et al., 2003; Gangalum et al., 2011). These studies of cargos sorting mechanisms can provide strategies for disease treatment by intervening the process of cargos loaded into exosome s, or develop more efficient exosome-mimics as a novel tool for drug delivery.

### Cargos in Exosomes

The role of exosomal nucleic acids, especially the ncRNA, has attracted more and more attentions. At present, the most extensive areas of research are mainly focused on three parts. First, exosomal ncRNA as diagnostic biomarkers for disease. Of note, exosomal ncRNA has the advantage as diagnostic biomarkers since exosomes exist in various types of human body fluids such as blood, urine, saliva, which can be easily obtained in clinic. For example, the upregulation of miRNA-139-3p in plasma exosome can predict the occurrence of colorectal cancer (Liu et al., 2020). In urine exosomes, the expression of miR-194-5p was significantly down-regulated in patients with autosomal dominant polycystic kidney disease (Magayr et al., 2020). In tear exosomes, the expression of miR-21 and miR-200 of patients with metastatic breast cancer was higher than that of healthy volunteers (Inubushi et al., 2020). Second, exosomal ncRNA participate in cell-to-cell communication through receptor-ligand interactions with target cells, promoting pathologic process such as inducing abnormal angiogenesis (Conigliaro et al., 2015), regulating immune response (Xu Z. et al., 2018), and enhancing chemical resistance (Xu et al., 2016). Third, exosomal ncRNA has been recently reported with a therapeutic role. Marleau et al. (2012) reported that pathogenic exosomal ncRNA can reduce the number of exosomes by inhibiting its sorting pathway or by blood filtration. Based on the sponge function of circRNA, artificial circRNA sponges loaded with exosomes have been designed to target pathogenic miRNA in diseases (Geng et al., 2020).

As another cargo category in exosomes, proteins can actually provide more direct information about disease progression. Interestingly, exosomes of different cellular origin share certain common protein components, such as tetraspanins (CD63, CD9, CD81, and CD82), integrins proteins, membrane fusion proteins, and several molecular chaperones (HSP70 and 90) (Pegtel and Gould, 2019). Like the exosomal ncRNAs in disease diagnosis, exosomal proteins have been demonstrated with higher sensitivity, specificity, and stability than traditional serum markers. In breast cancer patients, more than 100 phosphoproteins in plasma exosome were detected with diagnostic value (Chen et al., 2017). In cancer research, Glypican-1 (GPC1) in exosomes effectively helped to identify pancreatic cancer patients (Melo et al., 2015). Besides, by transporting functional proteins, exosomes can also participate in the progression of disease. Recently, exosomal PD-L1 has been indicated to mediate immune escape of tumor cells (Ricklefs et al., 2018); exosomal clusterin can improve myocardial performance and protect cell survival in mouse myocardial infarction model (Foglio et al., 2015); and inhibition of exosome MCP-1 expression can effectively protect retinal cell activity (Yu et al., 2016). By modifying the exosome surface protein by genetic engineering (Liu and Su, 2019), the exosome acts as a therapeutic tool. For example, exosomes with hyaluronidase PH20 on their membrane can activate dendritic cells (DC) to promote anti-cancer immune response (Hong et al., 2019). Another example is that exosome with high expression of MHC class I/peptide complexes can effectively activate CD8 (+) T cells (André et al., 2004).

In addition to constituting exosomal membranes, specific lipids (e.g., cholesterol, phospholipids, and phosphatidylserine) have been investigated and enriched in exosomes compared to their parent cells (Skotland et al., 2017). Notably, there are several similarities between the lipid composition of exosomes, HIV particles, and detergent resistant membranes (DRMs), isolated from cells as models for lipid rafts (Llorente A. et al., 2013; Lorizate M. et al., 2013). The first study of lipid biomarkers in exosomes was performed with urinary samples from eight patients with renal cell carcinoma and eight healthy volunteers (Lorizate M. et al., 2013). Subsequently, such studies have been reported on the diagnosis of prostate cancer (Clos-Garcia et al., 2018). To our knowledge, there has been no research on exosomal lipid as diagnosis biomarker for retinal diseases.

For exosomal small molecular substances, most studies have focused on the loading of chemotherapeutic drugs in exosome, such as incubation of paclitaxel with mesenchymal stem cell (MSC)-derived exosomes to inhibit tumor growth (Pascucci et al., 2014), catalase loading into exosomes to treat Parkinson’s disease (PD) (Haney et al., 2015).

## Exosomes in Retinal Disease

Although exosomes have been intensively investigated in cancer and cardiovascular diseases, its role in retinal diseases has not been fully studied. Owing to the relatively small volume vitreous and aqueous humor can be obtained, and for ethic issues, most research is still at the stage, mainly performed *in vitro*. On the other hand, retina, as the immune-privileged site, which is protected from external and internal insults by its blood-retina barriers and immune suppressive microenvironment, is actually an ideal target tissue for exosome-based therapies (Nam et al., 2020).

Under pathologic conditions, inflammation, oxidative stress, immune response, etc., exosomes can be generated by retinal cells, and secreted into extracellular spaces or vitreous cavity (Fukushima et al., 2020). In addition, the exosomes from remote cells or hematopoietic cells in patient with systematic diseases can also exert possible harmful effect on retina (Aires et al., 2020). Table 2 summarized the studies concerning the exosomes in retinal diseases.

**TABLE 2:** A selective overview of studies reporting exosomes in retina diseases.

**References**	**Origin of exosomes**	**Disease involved**	**Exosomal cargo**	**Biological function**	**Year of publication**
Inoue et al. (2007)	Mouse bone marrow MSCs	RD	NA	Delay of the photoreceptor apoptosis	2007
Hajrasouliha et al. (2013)	Retinal astroglial cells	Laser-induced CNV	Endostatin, CXCL-1, MIP-1α, MMP MMP-3, MMP-9, Nov, PEDF	Inhibition of CNV	2013
Ragusa et al. (2015)	Vitreous humor and serum	Uveal melanoma	miR-21, miR-34a, miR-146a	Promotion the proliferation of tumor cells	2015
Yu et al. (2016)	Mouse adipose MSCs and human umbilical cord MSCs	Retinal laser injury	NA	Down-regulation of MCP-1 and alleviation of laser induced retinal injury.	2016
Kannan et al. (2016)	RPE	AMD	αB-Crystallin	Neural protection for adjacent RPE cells and photoreceptors.	2016
Knickelbein et al. (2016)	ARPE-19	RD	Proinflammatory cytokines	Inhibition of T cell proliferation	2016
Mead and Tomarev (2017)	Human bone marrow MSCs	Optic nerve crush	NA	Neuroprotection and neurogenesis	2017
Shigemoto-Kuroda et al. (2017)	MSCs	Type 1 diabetes and experimental autoimmune uveoretinitis	TSG-6	Inhibition of the activation of antigen-presenting cells and inhibition of the development of T helper 1 (Th1) and Th17 cells	2017
Huang et al. (2018)	Plasma	DR	igG	Promotion of microvascular damage	2018
Safwat et al. (2018)	Rabbit adipose MSCs	DR	miR-222	Reduction of retinal degeneration	2018
Zhang et al. (2018)	Human umbilical cord MSCs	Macular holes		Promotion of the functional and anatomical recovery of macular hole	2018
Zhang et al. (2019b)	ARPE-19 under stress	AMD	IL-1β, IL-18, and caspase-1	Upregulation of NLRP3 inflammasome	2019
Liu et al. (2019)	Human retinal pericytes (ACBRI-183)	DR	cPWWP2A	Regulation of retinal microvascular function by Mir-579/ingiogenin 1/occludin/SIRT1 pathway	2019
Liu et al. (2019)	Human umbilical cord MSCs	Hyperglycemia-induced retinal inflammation	miR-126	Down-regulation of HMGB1 expression and NLRP3 inflammasome in HRECs induced by high glucose	2019
Mathew et al. (2019)	Human bone marrow MSCs	Retinal ischemia-reperfusion	NA	Enhancement of the functional recovery and reduction of the neuroinflammation and apoptosis	2019
Zhang et al. (2019a)	Platelet-rich plasma	DR	CXCL10	Upregulation of TLR4 signal pathway and mediation of hyperglycemia-induced retinal endothelial injury	2019
Ma et al. (2020)	Rat bone marrow MSCs	Retinal detachment	NA	Reduction of the expression of TNF-α and IL-1β and Inhibition of photoreceptor apoptosis	2020
Bian et al. (2020)	Neural stem/progenitor cell	RD	17 miRNAs (let-7a-5p, miR-26a-5p, miR-21-5p, etc.)	Protection of photoreceptor cells by inactivating microglia cells	2020
Nicholson et al. (2020a)	RPEs	AMD	Ligands and neuraminidase	Promotion of rapid absorption of receptor cells	2020
Zhang W. et al. (2020)	Platelet-rich plasma	DR	NA	Activation of YAP, enhancement of both the proliferation and fibrogenic activity of Müller cells via the PI3K/Akt pathway.	2020
Zhu et al. (2020)	Retinal astrocytes	DR	NA	Promotion of the proliferation and migration of endothelial cells	2020
Yang et al. (2020)	ARPE-19	AMD	NA	Aggravation of oxidative stress damage in RPE cells	2020
Ahn et al. (2020)	ARPE-19	AMD	miR-494-3p	Potential biomarkers of mitochondrial dysfunction	2020
Ke et al. (2020)	ARPE-19	AMD	Apaf1	Induction of inflammation and apoptosis in normal RPE cells via Apaf1/caspase-9 axis	2020
Fukushima et al. (2020)	ARPE-19	AMD	Angiogenic factors	Promotion of the angiogenesis	2020
Morris et al. (2020)	RPE	AMD	miR-21	Regulation of microglia function	2020
Seyedrazizadeh et al. (2020)	Human embryonic stem cells	Optic nerve injury	NA	Protection for retinal ganglion cells	2020
Aires et al. (2020)	Microglia	Glaucoma	NA	Activation of microglia and induction of cell death	2020
Gu S. et al. (2020)	ARPE-19	DR	miR-202-5p	Inhibition of growth, migration and tube formation of HUVEC cells	2020
Koh et al. (2020)	Human placenta-derived MSCs	Optic nerve injury	NA	Restore the expression of regeneration markers in R28 cells injured by hypoxia damage	2020
Zhang Y. et al. (2020)	ARPE-19	DR	miR-543	Induction of EMT in receptor RPE cells	2020
Nicholson et al. (2020b)	ARPE-19	AMD	Fibronectin, annexin A2	Trigger loss in transepithelial resistance (TER) in recipient monolayers mediated by HDAC6	2020
Yan et al. (2020)	Human 293T cells	Retinal ischemia	NA	Neuroprotective effect	2020
Gu C. et al. (2020)	Adipose MSCs	DR	miR-192	Negative regulation of ITGA1	2020
Li et al. (2021)	Human umbilical cord MSCs	CNV	miR-27b	Inhibition of EMT and subretinal fibrosis by targeting HOXC6	2021
Wang et al. (2021)	ARPE-19	RD	NA	Protect the photoreceptor and inhibit the expression of inflammatory factors, reduce the oxidative damage.	2021

***RD**, *retinal degeneration;****CNV***, *choroidal neovascularization;****AMD***, *age-related macular degeneration;****DR***, *diabetic retinopathy*;***MSCs***, *mesenchymal stem cell;****NA***, *not available, not mentioned in the article;****RPE***, *Retinal Pigment Epithelium;****EMT***, *epithelial-mesenchymal transformation.**

### Age-Related Macular Degeneration

Age-related macular degeneration (AMD) is the leading cause of vision loss in the elderly in Europe and the United States. The disease mostly occurs at the age of about 50 years old. With the older the age, the higher the incidence is. The pathogenesis of early AMD is characterized by the physiological phagocytosis of outer segments by retinal pigment epithelium (RPE) cells and the accumulation of excretion of residual metabolites from RPE cells to form drusen. Some studies have shown that the exosome surface proteins such as CD63, CD81, and LAMP2 expressed in the drusen of AMD patients (Wang et al., 2009). Meanwhile, it was also found that CD63 was co-located with the common proteins in drusen, suggesting that exosomes may co-excreted along with other metabolites from RPE cells. Ebrahimi et al. (2014) found that exosome participates in the release of complement regulatory factor RPE cells, such as CD46 and CD59, and this dysregulation the complement system plays important roles in the development of AMD. Photoreceptor cell death and microglia activation are also known to occur progressively in dry AMD pathogenesis. There is an inverse correlation between exosome concentration and photoreceptor survivability, with a decrease of exosome numbers following photoreceptor damage (Wooff et al., 2020). Using co-culture of RPE and retinal microglia, Morris et al. (2020). demonstrated that miR-21 was transferred from RPEs to microglia, which can further influence the expression of genes downstream of the p53 pathway in microglia.

αB crystallin is a small heat shock protein (HSP), which is known to be expressed in a variety of tissues *in vivo*. In retina, αB crystallin mainly exists in mitochondria and cytoplasm of RPE cells and participates in neuroprotection and regulation of angiogenesis (Kase et al., 2010; Kannan et al., 2012). Kannan et al. (2016) showed that αB crystallin can protect RPE cells from oxidative damage. The αB crystallin is also a regulator of angiogenesis and vascular endothelial growth factor (Kase et al., 2010). However, under pathological conditions, PRE cells can secrete exosomes loaded with αB crystallin, which further and promote the formation of drusen (De et al., 2007).

Oxidative stress plays important role in RPE proinflammatory damage. Nod–like receptor protein 3 (NLRP3) inflammasome has the capability to sense the reactive oxide species (ROS) produced by mitochondrial dysfunction, and further to activate caspase-1, promoting the precursor maturation of inflammatory cytokines IL-1 β and IL-18. Tarallo et al. (2012) and Kim et al. (2014) reported the activation of NLRP3 in RPE cells from patients with AMD. On the basis of this, Zhang et al. (2019b) found that the expression of IL-1 β, IL-18, and caspase-1 was up-regulated in exosomes isolated from APRE-19 under photooxidative blue-light stimulation, which demonstrated the role of exosomes in the regulation of NLRP3 inflammatory bodies. On the other hand, oxidative stress can not only promote the release of exosomes from RPEs, but also make receptor cells engulf exosomes more quickly in the progression of AMD disease (Nicholson et al., 2020a; Yang et al., 2020). Exosomes under oxidative stress can also increase apoptosis of ARPE-19 cells through overexpression of Apaf1 and induce oxidative damage and inflammation through caspase-9 apoptosis pathway (Ke et al., 2020). In another study exosome from J-cybrids reduced transepithelial resistance (TER) in receptor ARPE-19 cells, and this is associated with high risk of AMD (Nicholson et al., 2020b). Similarly, the mitochondrial damage of ARPE-19 could lead to an increase in the level of miR-494-3p in exosome (Ahn et al., 2020), which may be helpful for early diagnosis of AMD.

In wet AMD, abnormal choroidal neovascularization (CNV) is the hallmark that sprouts from the choroid vasculature and grows beneath and into the retina. Recently, Fukushima et al. (2020) reported that epithelial-mesenchymal transition (EMT) related factors can promote RPE cells to secrete exosome enriched of angiogenic factors, thus promoting the progress of CNV. On the contrary, the miR-27b derived from human umbilical cord mesenchymal stem cells can inhibit EMT by targeting HOXC6, thus delaying the progress of CNV (Li et al., 2021).

### Glaucoma and Optic Nerve Crush

Glaucoma is a disease characterized by optic papilla atrophy, visual field defect, and vision loss. The main mechanism of glaucoma is the chronic death of retinal ganglion cell (RGC), which is driven by many factors, such as high intraocular pressure. Reported pathological processes involved in RGCs death included neurotrophic factor deprivation, toxic pro-neurotrophins, mitochondrial dysfunction, activation of apoptotic signals, oxidative stress, and loss of synaptic connectivity (Almasieh et al., 2012). Exosome has also recently been indicated to play an important role in the process of glaucoma. Aires et al. (2020) reported that exosomes produced by retinal microglia under high intraocular pressure can propagate the inflammatory signals and promote retinal degeneration. Another two groups found that exosome derived from human placental MSCs and human 293T cells which express brain-derived neurotrophic factor (BDNF) can protect R28 cells (retinal precursor cells that represent RGCs) from hypoxia injury (Koh et al., 2020; Yan et al., 2020).

Optic nerve crush, as another animal model of glaucoma, is accompanied by the injury of retinal ganglion cells and axons. Previous studies have shown that MSC can effectively delay the apoptosis of RGC (Inoue et al., 2007). In a recent study, Mead and Tomarev (2017) isolated and injected MSCs-derived exosomes into the rat vitreous and showed that the exosomes successfully transported its neuroprotective cargos into RGC cells in maintaining the retinal function. Similarly, exosome derived from umbilical cord mesenchymal stem cells (UMSCs) and human embryonic stem cells (ES-MSCs) have also been found to yield similar neuroprotective effects (Pan et al., 2019; Seyedrazizadeh et al., 2020). In addition, Tassew et al. (2017) found that exosomes derived from fibroblasts can promote axonal regeneration by activating autocrine Wnt10b-mTOR pathway. These studies have collectively shown the therapeutic potential of exosomes in glaucoma and optic neuropathy.

### Diabetic Retinopathy (DR)

Diabetic retinopathy is one of the most common microvascular complications of diabetes mellitus. Vascular endothelial growth factor (VEGF) increases the permeability of retinal capillaries and macular edema, deteriorating the visual acuity. Under physiological condition, there is a balance between pro- and anti-angiogenic components in the eye. Hajrasouliha et al. (2013) found that exosomes released from retinal cells contain pro-angiogenic components, while exosomes from retinal astroglia cells (RACs) contain anti-angiogenic components. However, under oxidative stress, the exosomes released by RACs can promote the proliferation and migration of endothelial cells (Zhu et al., 2020). Similarly, exosomes derived from platelet-rich plasma (PRP-Exos) can also mediate retinal endothelial injury by up-regulating TLR4 signaling pathway (Zhang et al., 2019a). Besides, PRP-Exos can also promoted the proliferation and fibrosis of Müller cells (Zhang W. et al., 2020). As endothelial-to-mesenchymal transition (EndoMT) being implicated in endothelia dysfunction in DR, Gu S. et al. (2020) found that high glucose (HG)-induced miR-202-5p in exosomes of ARPE-19 cells had a negative regulatory effect on the expression of transforming growth factor β2 and inhibited HG-induced EndoMT.

As one of the main cells that make up retinal blood vessels, pericytes, if lost, early damage of the vessel occurs (Hammes et al., 2002). Liu et al. (2019) showed that diabetes-related stress can up-regulate the expression of cPWWP2A in pericytes. The high level of cPWWP2A loaded within the exosomes can be transferred from pericytes to endothelial cells, leading to the angiogenic alteration of the receptor cells (Liu et al., 2019).

The activation of complement system has also been implicated in the dysfunction of blood-retinal barrier. As reported, the plasma IgG-laden exosomes could activate the classical complement pathway and the quantity of these exosomes was increased in diabetes (Huang et al., 2018). Compared with diabetic mice, STZ-induced diabetic mice that lack the expression of IgG resulted in less retinal vascular leakage (Huang et al., 2018). Further, plasma IgG-laden exosomes has been demonstrated to lead to membrane attack complex (MAC) deposition and contribute to retinal endothelial damage by activating the classical complement pathway (Huang et al., 2020).

The therapeutic potential of exosomes in DR has also been a great concern for researchers. Exosome of adipose MSCs containing miR-192 or miR-222 can suppress the inflammatory response and angiogenesis of DR (Safwat et al., 2018; Gu C. et al., 2020). In another research (Zhang et al., 2019c), the researchers injected UMSC-Exos with overexpression of miR-126 into the vitreous of diabetic rats, and found that those exosomes successfully inhibited HMGB1 signaling pathway and alleviated inflammation induced by hyperglycemia.

### Autoimmune Uveitis

Autoimmune uveitis, mainly mediated by antigen-specific T lymphocytes including Th1 and Th17 cells, is an autoimmune disease involving a number of inflammatory cytokines. Shigemoto-Kuroda et al. (2017) found that MSC-exos can effectively suppress the immune response in mice with experimental autoimmune uveoretinitis (EAU). Compared with the control group injected with PBS, pro-inflammatory cytokines, IFN-γ+CD4+cells, and IL-17+CD4+cells were significantly decreased in MSC-exos injection group. On the other hand, Knickelbein et al. (2016) demonstrated that exosomes produced by RPEs and peripheral blood monocytes from patients with non-infective uveitis could significantly inhibited the proliferation of T cells. In EAU mice model, Kang et al. (2020) found that exosomes derived from IL-35-producing regulatory B-cells can induce the expansion of IL-10-producing T cells (Tregs), and IL-35-producing T cells (iTR35), alleviating the autoimmune uveoretinitis in mice (Dambuza et al., 2017). In clinic, Zheng et al. (2020) conducted a proteomic analysis of exosomes in patients with Vogt-Koyanagi-Haradasyndrome (VKH) disease and found that the levels of carbonic anhydrase 2 and RAS-related protein Rap-1b can be used as biomarkers to predict active inflammation of VKH disease. However, further studies are needed to investigate how the exosomes from the body provoke the ocular autoimmune pathogenesis.

### Other Retinal Diseases

Retinal laser injury is a kind of thermal injury that directly burns the retina. In a mouse model of retinal injury induced by laser, intravenous injection of MSC can inhibit retinal cell apoptosis (Jiang et al., 2014). Further experiments showed that monocyte chemotactic protein (MCP)-1 in retina was significantly down-regulated by intravitreal injection of MSC-exos, and both MSC-exos and MSC could reduce apoptosis of retinal cells. At the same time, blocking the expression of MCP-1 can effectively protect the viability of retinal cells, indicating that MSC-exos can reduce retinal injury by targeting MCP-1 (Yu et al., 2016).

Macular hole (MH), which is defined as a full-thickness defeat in the neuroretina of the macula. Vitrectomy with ILM peeling or inverting is the main method for anatomical reduction, but the functional recovery is not satisfactory. Few literatures on the treatment of macular holes with stem cells-derived exosomes can be obtained. Zhang et al. (2018) investigated the efficacy of postoperative injection of MSC (*n* = 2) or MSC-exos (*n* = 5) in the treatment of seven patients with refractory macular hole. The results showed that the BCVA of five closed-hole eyes improved after operation. Compared with MSC-exos injection, one patient with MSC injection showed a epiretinal fibrous membrane. However, the limitation of this study lies in the small number of patients and the control group, and further research is needed to evaluate the actual therapeutic effect.

Retinal detachment (RD) is defined as the separation of the neurosensory retina from the RPEs. Ma et al. (2020) found that MSC-Exos have therapeutic effects on reducing retinal inflammation, enhancing autophagy, and inhibiting photoreceptor cell apoptosis in a rat RD model. Further exosomal proteins analysis identified a total of 683 independent proteins, among which 9 proteins that may play anti-inflammatory, neuroprotective and anti-apoptotic effects in this RD model.

Ocular malignant tumor is a life-threatening disease, usually because of its easy recurrence, and high rate of metastasis. In the past few years, the role of exosomes in cancer field has been much stressed. The exosomes can not only participate in tumor angiogenesis (Zeng et al., 2019), but also promote tumor growth and tumor metastasis (Biswas and Acharyya, 2020). In the research of Eldh et al. (2014), the exosomes isolated from the liver perfusion fluid of patients with metastatic uveal melanoma were found to carry positive melanoma-specific marker Melan-A, indicating that there were exosomes of tumor origin in the liver perfusion. In addition, Ragusa et al. (2015) found that compared with the control group, there were upregulated expressions of miR-21, -34a, -146a in vitreous and vitreous exosomes, and there were upregulated miR-146a in serum and exosomes of UM patients. In a more recent study, the authors performed proteomic analysis of exosomes derived from retinoblastoma and identified that several proteins were involved in extracellular matrix (ECM) remodeling, and the metabolism/catabolism of glucose and amino acids (Galardi et al., 2020). These two studies make it an expectant method to diagnose eye tumor by collecting periocular tissue or body fluid samples and detecting the specific exosomes.

## Exosomes as Therapeutic Agents

**FIGURE 2:**
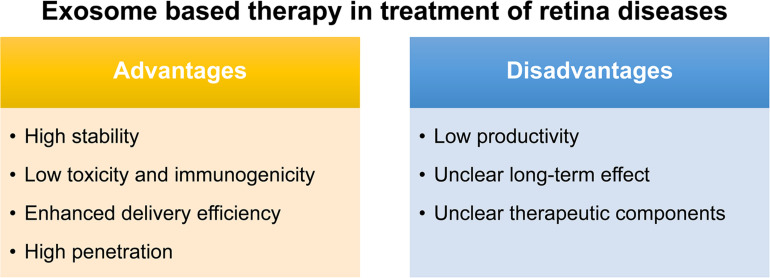
Advantages and disadvantages of exosome-based therapy in treatment of retinal diseases.

The conventional route of treatment for eye disease usually needs the frequent administration of topical instillation of eye drops which has low bioavailability. Although various synthetic drug vehicles have been recently developed for encasing existing drugs to enhance the therapeutic effect (Kompella et al., 2020), troubling issues should keep in mind including their immunotoxicity (Pandey and Prajapati, 2018) and quick clearance by the mononuclear phagocyte system or the reticuloendothelial system (Haque et al., 2016). Fortunately, exosomes, reported as natural nanocarriers to deliver drugs/nucleic acids, providing several. First of all, exosomes are naturally present in body fluids so that they are stable under both physiological and pathological conditions. As reported, immune related miRNA derived from human breast milk are shown to be very stable in very acidic conditions, thus tolerating an infant’s gastrointestinal environment (Kosaka et al., 2010). Second, exosomes are less toxic and immunogenic compared to other carriers. For example, both viral and non-viral carriers have been developed as gene delivery vehicles in retinal diseases, however, they have major drawbacks such as a high systematic toxicity and trigger immune response (Verdera et al., 2020). Compared with intravitreal injection of traditional adeno-associated virus 2 (AAV2), the novel exosome-associated AAV2 (exo-AAV2) revealed that exo-AAV2 carrying the GFP-coding gene AAV was more strongly expressed and had greater penetrability in the retina (Wassmer et al., 2017). Compared with MSCs themselves used to treat various diseases, exosome-based therapeutics have a lower risk for teratoma formation and embolization, which are major concerns for stem cell-based therapeutics (Shigemoto-Kuroda et al., 2017; Pan et al., 2019; Seyedrazizadeh et al., 2020). Third, due to unique membrane proteins and lipids that bind to specific receptors on the recipient cell surface, exosomes are driven to deliver cargo to specific recipient cells, hence, enhancing the delivery efficiency (Alvarez-Erviti et al., 2011). Finally, exosomes are able to penetrate the biological barriers including the blood-brain barrier (BBB) and the blood-retina barrier (BRB), a major challenge in systemic drug delivery research as most drugs and carriers cannot cross this barrier (Elliott and He, 2021; Figure 2).

Inspired by these advantages and their ability to transfer regulatory molecules, investigators are developing several strategies for the treatment of retinal diseases via exosomes. Hajrasouliha et al. (2013) reported that the exosomes of retinal astrocytes can reduce vascular leakage and inhibit experimental CNV. The exosome of PRE cells can regulate immune response and protect photoreceptor cell (Knickelbein et al., 2016; Wang et al., 2021). In a recent research, exosomes derived from neural stem/progenitor cells (NPCs) were injected into the subretinal space of rats with retinal degeneration, and those NPC-exos were found to significantly inhibit the activation of microglia and protect photoreceptors from apoptosis (Bian et al., 2020). Exosomes from stem cells, such as MSCs, can also play an important role in improving retinal laser injury (Yu et al., 2016), high glucose-induced retinal inflammation (Zhang et al., 2019c), and inhibiting the migration of EAU inflammatory cells (Bai et al., 2018). Similarly, The exosomes of human BMSCs also show promising therapeutic potential in neuroprotection (Mead and Tomarev, 2017), improvement of retinal ischemia (Mathew et al., 2019), and protection from photoreceptor apoptosis (Bian et al., 2020).

Notably, exosomes as a therapeutic agent also have some issues to be overcome. Firstly, a great difficulty for their translation to the clinic would be the low productivity of exosomes obtained with almost all the isolation methods, and also the presence of some impurities such as proteins or other extracellular vesicles. Secondly, though the exosome plays certain therapeutic role, most of their therapeutic function were demonstrated *in vivo* and the long-term effect is uncertain. Thus, how to demonstrate and maintain the long-term therapeutic effect is also an urgent problem to be solved. Thirdly, the key components which function were not identified in most stem-cell based exosome studies (Mead and Tomarev, 2017; Greening and Simpson, 2018; Huang et al., 2018; Zhang et al., 2018; Mathew et al., 2019; Pan et al., 2019; Ma et al., 2020; Figure 2). To identify the key components in therapeutic exosomes might be helpful for the preparation of exosomes with more efficiency. Besides, in animal models, the exosome is usually injected into the vitreous or subretinal space to explore its therapeutic effect, but there is still a long way to go for the exploration of the relationship between the best therapeutic effect and the injection way, the frequency, and the dose. In the future, more in-depth research can be done on the way of drug administration of the exosomes.

## Conclusion

Taken together, exosome research in the eye field is trailing other fields in exosome research, awaiting more extensive investigations to dedicate exosomes in retinal pathogenic process and their attractive diagnostic and therapeutic roles in retinal diseases. However, there is still a long way to the successful translation into clinical therapies before novel technology for mass highly purified exosomes with stable long-term functional efficacy, and elucidation of the key components of exosomes and understanding how each of them. Anyway, we believe that with the future in-depth study of exosomes and the development of novel technologies in retinal diseases, we can have a better understanding of the role of exosome in retinal pathogenesis. For patients, the progress of exosome research can be very promising in providing more personalized treatment and diagnosis, so as to shed light on new therapy on treating retinal diseases for years to come.

## Author Contributions

ZZ, PX, and ZH mainly performed the literature research and drew and revised the manuscript. AM drew the figures and prepared the tables. AF and QL contributed to the discussion and manuscript review. All authors contributed to the article and approved the submitted version.

## Conflict of Interest

The authors declare that the research was conducted in the absence of any commercial or financial relationships that could be construed as a potential conflict of interest.
